# The Impact of Temperature on the Sporogonic Development of the Tropical Avian Malaria Parasite *Plasmodium relictum* (Genetic Lineage pGRW4) in *Culex pipiens* Form *molestus* Mosquitoes

**DOI:** 10.3390/microorganisms9112240

**Published:** 2021-10-28

**Authors:** Elena Platonova, Vaidas Palinauskas

**Affiliations:** 1 Nature Research Centre, Akademijos 2, 08412 Vilnius, Lithuania; 2 Biological Station Rybachy, Zoological Institute, Russian Academy of Sciences, 238535 Kaliningrad, Russia

**Keywords:** avian malaria, *Plasmodium relictum*, pGRW4, transmission, sporogony, *Culex pipiens*, vector

## Abstract

The avian malaria parasite *Plasmodium relictum* (genetic lineage pGRW4) is known to cause severe pathology in nonadapted vertebrate hosts. This parasite is prevalent in some bird species in Northern Europe, however the records obtained are only from adult long-distance migrant birds after their return from the wintering grounds. A recent experimental study showed that this parasite completes sporogonic development in the local European vector *Culex pipiens* at a controlled mean temperature of 19 °C. Thereby, temperature limits for the transmission of this parasite in Northern Europe remain unknown. In this study, we took a step further and tested the impact of different temperature conditions, including some extreme fluctuations between 23 °C down to 7 °C, on the sporogonic development of *P. relictum* (pGRW4) in the vector *Culex pipiens* form *molestus*. Mosquitoes were exposed to infection and kept under different air-temperature conditions: (i) constant warm temperature, (ii) natural outdoor temperatures and (iii) temporary exposure to low temperatures. *Plasmodium relictum* (pGRW4) completed sporogony in mosquitoes of all experimental groups, however different patterns of the sporogonic development depending on temperature conditions were observed. Based on these results, we conclude that the cool air temperature of Northern Europe in summer is not a limiting factor in successful development of the parasite. However, delayed sporogony caused by low summer temperatures may have a detrimental impact on the active transmission of this parasite in Northern Europe.

## 1. Introduction

The spread of parasitic pathogens into new geographical areas poses a potential risk for native animal populations, as novel parasites could be highly virulent to the local hosts that have not coevolved with the new arrival [1,2,3]. The negative impacts of the newly introduced and then established parasites are documented in wild populations, captive and domestic animals, and livestock [4,5,6,7].

Avian haemosporida are apicomplexan parasites with a heteroxenous lifecycle, whose definitive hosts are blood-sucking dipteran insects and intermediate hosts are birds [5,8]. These parasites are widely distributed and are able to infect numerous bird species from different taxa [9,10,11]. Avian haemosporidian parasites are known to cause severe pathologies in naïve hosts and can potentially lead to a decline of wildlife populations [12,13,14,15,16,17].

In Europe, including the northern temperate regions, transmission of various *Plasmodium* species is common [5,18,19,20]. Former studies have revealed the presence of tropical malarial parasites in blood of the long-distance migrants after their return from their wintering grounds in Africa and South Asia [20,21,22]. Some of these *Plasmodium* parasites are highly virulent to European bird species [23,24,25,26]. The tropical haemosporidians are not transmitted in the temperate zone of Europe, although, the possibility to complete their development in local vectors (i.e., sporogonic development) or avian hosts has been demonstrated [24,26,27,28,29]. *Plasmodium relictum* (pGRW4) infects more than 80 bird species and has transmission mostly in tropical regions and the Americas [30]. This parasite causes severe malaria in new avian hosts and is particularly notorious for having caused the extinction of some native bird species on the Hawaiian Islands [31], and likely in Cuba [32], where this parasite was introduced. *Plasmodium relictum* (pGRW4) is brought to Europe annually by birds wintering in tropical Africa, mainly by passerines of the genus *Acrocephalus* [11]. The prevalence of this parasite is relatively high (reaching 15–20%) in adult Great reed warblers *Acrocephalus arundinaceus* in northern regions during the summer [21,33,34]. This makes this species one of the most important sources of pGRW4 in Europe. Great reed warblers arrive to breeding sites in Northern Europe approximately in the middle of May and migrate from their breeding sites in early August [35]. The screening of blood smears obtained from warblers during the breeding season shows that the parasite is circulating in blood of infected birds, and thus, infection of susceptible vectors is possible [23].

*Culex quinquefasciatus* is the main vector of pGRW4, but this mosquito is absent in Europe [30]. It has been proven that *P. relictum* (pGRW4) is capable of completing development in the European mosquito *Culex pipiens* under laboratory conditions [28,36]. This species is widely distributed across the world, common and abundant in northern regions of Europe [37], and is known as one of the main vectors of avian malaria parasites among local birds [28]. *Culex pipiens* transmits, inter alia, *Plasmodium relictum* genetic lineage pSGS1 which is closely related to pGRW4, but despite great efforts, pGRW4 has never been found to be transmitted in the temperate climate regions in Europe [28].

Ambient temperature is considered to be one of the main factors influencing the development of malaria parasites in their vectors [5,38,39]. The pronounced detrimental effects of low temperature on sporogonic development are well known in different species of malaria parasites [9,39,40]. A recent experimental study conducted by Valkiūnas et al. [28] showed that *P. relictum* (pGRW4) completes sporogony in *C. pipiens* f. *molestus* mosquitoes at a relatively low mean temperature of 19 °C (ranging between 14 °C at night and 24 °C during the day). Based on these data, the authors concluded that the temperatures used in the study do not play a role in limiting the transmission of this parasite. However, in Northern Europe, for instance at our study site located in a temperate climate zone, the average temperature in June is 15 °C, with normal fluctuations and temperatures at night that can go down below 10 °C (annual measurements of temperature on the study site, unpublished data). These sporadic changes could be crucial for sporogonic development of the parasite [41]. The completion of sporogony may be prolonged to July or the beginning of August, when long-distance migrant birds, which are the main reservoir of exotic *Plasmodium* spp., leave the nests, and soon after start to migrate to the wintering grounds. Thus, this might cause a gap between the presence of the vector containing infective parasite stages and availability of susceptible vertebrate.

In our study, we test the assumption that the short-term cold snaps that are common in Northern Europe in June could serve as a limiting factor in *P. relictum* (pGRW4) sporogonic development, and therefore, restrict pathogen transmission in this region. We experimentally infected *C*. *pipiens* f. *molestus* mosquitoes to determine the sporogonic development of *P. relictum* (pGRW4) under different air-temperature conditions: (i) constant warm temperature, (ii) natural outdoor temperatures and (iii) temporary exposure to low temperatures.

## 2. Materials and Methods

### 2.1. Ethical Statement

The procedures of bird capture, collection of blood samples and the experimental infection of the birds and the mosquitoes were approved by the International Research Co-operation Agreement between the Biological Station “Rybachy” of the Zoological Institute of the Russian Academy of Sciences and Nature Research Centre (4 September 2015). All the procedures with animals mentioned herein were approved by the Ethical committee of Zoological Institute of Russian Academy of Science (permission number 2019-02-05-2/1).

### 2.2. Study Site and Design of the Experiment

The experimental study was performed at the Biological Station “Rybachy” (the Department of Zoological Institute of Russian Academy of Sciences) on the Curonian spit in the Baltic Sea (55°09′ N, 20°52′ E) from June to August 2019. *Culex pipiens* f. *molestus* mosquitoes were used as vectors of avian malaria parasites, because these blood sucking insects are prevalent in Europe and can serve as vectors of different *P. relictum* lineages. The laboratory colony of insects was established from the egg rafts obtained from the Nature Research Centre (Vilnius, Lithuania) in April 2019 and was maintained throughout the study.

Two juvenile Eurasian siskins, *Carduelis spinus* were used as donors for feeding and the experimental infection of mosquitoes. Birds were caught using mist nets, ringed and checked for the presence of haemosporidian infection. The blood was taken with a heparinized capillary after puncturing the brachial vein with a thin needle (27 G size). Approximately 40–50 μL of blood was placed in SET buffer (0.05 M Tris, 0.15 M NaCl, 0.5 M EDTA, pH 8.0) for molecular analysis (see below) and two blood smears were prepared for microscopy. Smears were dried, fixed in absolute methanol, stained according to the Romanowski–Giemsa protocol [42] and examined for the presence of blood parasites. Uninfected birds were selected for the experiment and kept in a vector-free vivarium under controlled conditions (temperature 21–23 °C, humidity 60–70%, light/dark photoperiod 17/7). Each bird was housed in a separate cage (60 × 40 × 40 cm, Joko GmbH, Syke, Germany); food and water were provided every day ad libitum.

Before the experiment, birds were experimentally infected with the avian malaria parasite *P. relictum* (pGRW4). The strain of the parasite was isolated in 2018 from an adult, wild-caught, Sedge warbler *Acrocephalus schoenobaenus*, multiplied in siskins and cryopreserved in liquid nitrogen for further experimental infection according to Dimitrov et al. [29]. In 2019, one blood sample with pGRW4 infection, frozen in liquid nitrogen, was obtained and used to infect one Reed warbler *Acrocephalus scirpaceus*. This bird species is phylogenetically closely related to Sedge warbler and is suitable for the first multiplication of the parasite. The cryopreserved sample was thawed, and approximately 0.1 μL of the mixture was inoculated in pectoral muscles of the recipient warbler. When parasitaemia developed in the exposed reed warbler, its blood was used to additionally infect two siskins. The donor blood was mixed with 0.9% saline and sodium citrate (in 4:5:1 proportion) and approximately 6 × 10^4^ meronts of *P. relictum* were inoculated into pectoral muscles of each recipient bird [43].

Every 4 days, the blood was collected for measuring the level of gametocytaemia. When gametocytaemia increased to 0.2% or higher, the infection of mosquitoes was performed. Gametocytaemia in donor birds varied between low (0.2–0.5%) and high (3–5%) intensities during mosquito infection. For exposure of control mosquitoes, we used an uninfected siskin as a donor. At the end of experiment, infected donor birds were euthanized.

### 2.3. Experimental Infection of Vectors and Dissection of Female Mosquitoes

Uninfected female mosquitoes were randomly separated from the main colony and placed in an insect cage (45 × 45 × 45 cm, BugDorm, Taipei, Taiwan) with an immobilized, infected donor siskin. The bird was fixed in a carton tube so that only its legs were accessible to the mosquitoes. The age of mosquitoes, which were used for infection, varied between 7 and 12 days after emergence. After 1 h, full-fed females were caught with an insect aspirator and randomly placed in three small nylon-net cages (17.4 × 17.5 × 17.5 cm). Collected mosquitoes were divided into 4 experimental groups. After infection, insects of the first experimental group (E1) were kept indoors with a stable day- and night-time average temperature of 23 ± 1 °C, with a controlled photoperiod. The second group (E2) of insects was maintained outside the biological station under local natural conditions (the average daytime temperature of 21 °C and average night-time temperature was 15 °C). The third group (E3) of mosquitoes was kept indoors at a daytime temperature of 23 ± 1 °C and a night-time temperature of 7 °C with the same photoperiod as the insects of E1 group. The fourth group (E4) of control mosquitoes was kept indoors under the same temperature and photoperiod conditions as the first group of mosquitoes. Mosquitoes were kept for 28 days post exposure (dpe) and were gradually dissected for the preparations of different sporogonic stages of the parasite. Pads of cotton moistened with 9% sucrose solution were provided to feed mosquitoes, and small cups with water were placed for oviposition. In total, 157 mosquitoes took a blood meal from donor birds (30 individuals in group E1, 54 in group E2, 48 in group E3 and 25 in group E4).

From day 6 after exposure until the end of the experiment, dissection of the exposed mosquitoes was carried out every two days according to Žiegytė et al. [44]. Oocyst preparations were made from female mosquitoes dissected 6–28 dpe and sporozoite preparations from those dissected 14–28 dpe. Weak (dying) mosquitoes were processed with human endpoint, dissected and included in the data analysis. We used the number of oocysts as a proxy for the start of sporogonic development and then the presence of sporozoites to prove the success of completion of sporogony in mosquitoes. All sporogonic preparations were made following the standard protocol [5,28,44]. Before dissection, mosquitoes were euthanized by short exposure to 96% ethanol vapours. To make an oocyst preparation, the midgut of the infected mosquito was extracted on a glass slide in a drop of normal saline and temporarily stained with 2% mercurochrome. The fresh preparation was covered with a coverslip and screened under the light microscope Olympus CH20 (magnification 40 × 10). The numbers of oocysts were counted in each preparation. Then, the midgut was gently rinsed in 10% formalin and a permanent preparation of oocysts was made according to Valkiūnas [5]. Salivary glands of the infected insects were extracted and ground on a glass slide in a small drop of saline to make sporozoites preparations. The crushed content of glands was smeared on a slide, dried, fixed in absolute methanol and stained following the same protocol as that for blood smears (see above). To determine the presence of sporozoites, preparations were screened under 100× magnification using an oil immersion objective [44]. After dissection, residual parts of the insects (the head, abdomen and thorax) were stored in 96% ethanol for the molecular confirmation of the parasite lineage.

### 2.4. Molecular Analysis

A molecular analysis was performed to confirm that experimental birds prior to infection were free of malarial parasites, and then to confirm that the donor birds used in this study and exposed mosquitoes were infected only with *P. relictum* (pGRW4), excluding the presence of extraneous haemosporidian infections. Total genomic DNA was extracted from bird blood and experimental mosquitoes using the ammonium-acetate extraction method [45]. A 478 bp fragment of the parasites’ mitochondrial cytochrome *b* gene was amplified following the nested PCR protocol [46]. The positive (DNA of *P. relictum*, genetic lineage pSGS1) and negative (nuclease-free water) control samples were used to control the correctness of amplifications. The final PCR-products were checked by running them in 2% agarose gel, purified and prepared for sequencing [46]. DNA fragments were sequenced from both 5′ and 3′ ends using an ABI PRISM TM 3100 capillary sequencing robot (Applied Biosystems, Foster City, CA, USA). The sequences obtained were assembled using the BioEdit software (v. 7.0.5.3, Raleigh, NC, USA) [47] and compared to those available in the MalAvi database [11].

### 2.5. Statistical Analysis

Before statistical analysis, the data on the numbers of *P. relictum* (pGRW4) oocysts were merged to four main groups following an example from the previous study [28]. The oocysts for the first group were counted from infected mosquitoes 6–11 dpe (the first days of oocyst development), for the second group 12–16 dpe (when intensities of oocysts in midguts of infected mosquitoes were the highest), for the third group 17–22 dpe (when the numbers of oocysts were decreasing), and for the fourth group 23–28 dpe (when oocysts were disappearing from the midgut). Statistical analysis was performed using the R software [48].

To compare numbers of oocysts developed from high and low parasitaemia, a Wilcoxon rank-sum test was used. The nonparametric Kruskal–Wallis H test (KWt) was applied to compare numbers of oocysts and mortality between the groups. To compare mortality between E1 and E4 groups, χ^2^ test was used. A *p*-value of 0.05 or higher was considered as not significant.

## 3. Results

The molecular analysis showed that the experimental mosquitoes were exposed to a single infection of *P. relictum* (pGRW4) and excluded the presence of extraneous haemosporidian parasites in the blood of donor birds. Although the donor birds with different gametocytaemia were used, there was no significant difference between numbers of oocysts in the mosquitoes exposed to the donors with higher (3–5%) and lower (0.2–0.5%) intensities of gametocytaemia in the blood (W = 1122.5, *p* = 0.1365; Figure S1).

**Figure 1:**
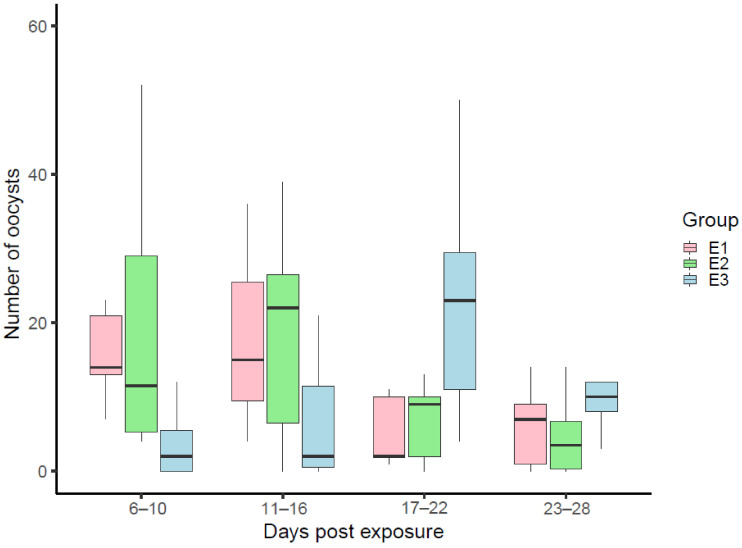
Numbers of *Plasmodium relictum* (pGRW4) oocysts in the *Culex pipiens* form *molestus* under different temperature conditions: (E1) indoors, stable day- and night-time temperature of 23 ± 1 °C; (E2) natural conditions outside the biological station (average daytime temperature of 21 °C and average night-time temperature of 15 °C); (E3) indoors, daytime temperature of 23 ± 1 °C and night-time temperature of 7 °C.

In mosquito midgut preparations of all experimental insect groups (E1–E3), oocysts were visible starting from 6 dpe, but the numbers of oocysts in mosquitoes of experimental group E3 were significantly lower (KWt: χ^2^ (2) = 10.711, *p* = 0.0047) and remained relatively low during 11–16 dpe but did not differ significantly comparing mosquitoes to those from E1 and E2 groups (KWt: χ^2^ (2) = 5.3070, *p* = 0.07) (Figure 1). During 17–22 dpe, the number of oocysts in mosquitoes of groups E1 and E2 decreased, while the mean number of developed oocysts in mosquitoes of E3 group reached a peak (Figure 1). During this period of sporogony there was a significant difference observed between experimental groups in the number of developed oocysts (KWt: χ^2^ (2) = 6.8773, *p* = 0.0321). During 23–28 dpe the numbers of oocysts decreased in mosquitoes of all experimental groups and did not differ statistically (KWt: χ^2^ (2) = 3.1816, *p* = 0.2032). Thus, the most intensive development of oocysts in mosquitoes of E1 and E2 groups was recorded during 6–16 dpe, with the highest peaks reached 11–16 dpe, while the peak of the mean oocyst numbers in E3 group mosquitoes was delayed and was recorded 17–22 dpe (Figure 1).

The examination of salivary gland preparations of the infected mosquitoes showed the presence of sporozoites in mosquitoes of all the experimental groups. In E1 group mosquitoes, sporozoites appeared 14 dpe and were observed until 26 dpe (89% of positive preparations). In E2 group mosquitoes, sporozoites were observed from 16 to 28 dpe (86% of positive preparations). In salivary glands of mosquitoes maintained at low temperatures (E3 group), sporozoites were observed only starting from 20 dpe until the end of the experiment (60% of positive preparations). No statistically significant difference was recorded in mosquito mortality between experimental groups. A total of 27% of mosquitoes died in group E1, 24% of mosquitoes in group E2 and 22% of mosquitoes in group E3. In the control group, 16% of mosquitoes died during the experiment. Mortality of infected mosquitoes in E1 group did not differ from uninfected controls (χ^2^ = 0.2, df = 1, *p* = 0.66).

## 4. Discussion

In the present study, we investigated sporogony of a tropical, avian malaria parasite under different temperature conditions. The obtained experimental results indicated that the avian malaria parasite *P. relictum* (pGRW4) completes sporogony in *C. pipiens* mosquitoes even when vectors are exposed to low (7 °C) night temperatures. However, lower temperatures were found to lengthen the development duration of oocysts and sporozoites.

The sporogonic development of avian malaria parasites at different temperatures was analysed in a number of studies previously [49,50,51,52,53,54]. It was experimentally shown that successful sporogony and the number of oocysts in the midgut of an infected mosquito depend on many physical and biological factors, including environmental temperature, humidity, and the simultaneous presence of vertebrate host, vector and parasite species in the same place [49,50,51,54,55]. According to earlier studies, the optimum temperature for *P. relictum* development in vectors is 25–27 °C, and temperatures of 10–20 °C may cease the sporogonic development [49,50,53,56,57]. However, it is unclear which particular lineages were used in these studies, as different genetic lineages belonging to the same morphospecies may have different infectiveness and different patterns of development. For example, genetic lineages pSGS1, pGRW4, pGRW11, pLZFUS01 and pPHCOL01 belong to *P. relictum* species and they differ in their host rage, geographical distribution and other biological characteristics [58,59].

A recent experimental study conducted by LaPointe et al. [41] showed that *P. relictum* from the Hawaiian Islands is not able to complete sporogony in *Culex quinquefasciatus* at temperatures lower than 17 °C, and the optimum temperatures are 28–30 °C. We assume that in the above-mentioned study, the pGRW4 lineage of *P. relictum* was used, as authors indicated that the isolate was local to Hawaii. Based on previous studies, pGRW4 is the only lineage of *P. relictum* circulating in Hawaii [60,61].

Interestingly, the sporogonic development time of *P. relictum* (pGRW4) on the Hawaiian Islands almost doubled and tripled at temperatures that were one or two degrees lower than 21 °C, respectively [41]. In our study, the duration of the sporogonic development in the mosquitoes kept outdoors, (exposed to daytime temperatures of 21 °C and night-time temperatures of 15 °C) was similar with that of the insects kept at a constant temperature of 23 ± 1 °C. The appearance of sporozoites in salivary glands of the Hawaiian *P. relictum* was recorded 11 dpe at a constant temperature of 20 °C and 21 dpe at a constant temperature of 16 °C. In our study, the first record of sporozoites varied between 14 dpe in E1 group mosquitoes, which were kept under a constant temperature of 23 °C and 20 dpe in the mosquitoes which experienced cold nights (E3). These results correspond to those reported by Ball and Chao [49], where *P. relictum* developed sporozoites that were first observed 20–22 dpe in *Culex tarsalis* kept at lower temperatures. Slight developmental differences between the Hawaiian and European/African pGRW4 lineages might be due to the different vector species (*C. quinquefasciatus* was used in the study by LaPointe et al. [41] and *C. pipiens* in the present study), or due to the isolation and evolved different biological features of the parasite. According to Valkiūnas et al. [58], different geographical isolates of pGRW4 show different biological features. For instance, after experimental infection of canaries, the Hawaiian isolate of pGRW4 can develop intensities of parasitaemia as high as 30% [62], while canaries infected with the European/African isolate of pGRW4 are resistant or show transient, light infections. Genetic differences between these pGRW4 isolates were also pointed out by Hellgren et al. [60]. According to these authors, the analysis based on the nuclear merozoite surface protein 1 (*MSP1*) gene showed that European/African isolates differ from those prevalent on the Hawaiian Islands. Genetic differences between these isolates may manifest in the developmental peculiarities both in vertebrate hosts and vectors.

Transmission of haemosporidian parasites in Northern Europe is limited and occurs only during the warm months of the year. Even during these months, especially in June, the daily temperatures can fluctuate and fall below 10 °C. Thus, the impact of cooler temperatures on sporogony might explain the absence of transmission of *P. relictum* (pGRW4) and other tropical parasites in Northern Europe despite the presence of suitable avian hosts and competent vectors. The results of the present study do not support the assumption that the tropical, avian malaria parasite *P. relictum* (pGRW4) is not able to complete sporogony in infected *C. pipiens* under natural conditions (E2 group) or even after exposure of experimental mosquitoes to a temperature of 7 °C every night (E3 group). The negative impact of the cooler temperatures on the parasite manifested in the duration of sporogony in experimental mosquitoes. In the preparations of midgut and salivary glands of the infected E3 group individuals, oocysts and sporozoites were observed after a significantly longer period than in those of groups E1 and E2. According to Ball and Chao [49], when mosquitoes are exposed to lower temperatures, not only do sporozoites appear in salivary glands later, but they also need additional time (5–28 days) for maturation to be infective after they arrive in the glands. This means that even when sporozoites are visible in salivary glands, more days of favourable temperatures are needed to finish maturation. These delays in development and maturity might be one of the key obstacles for successful transmission of some tropical malarial parasites in temperate climate zones. For example, during the breeding period, juvenile birds are the most vulnerable, as their immune system is immature, and they are confined to the nest where they may be infected with haemosporidian parasites from the vectors visiting the nest [5]. The relatively high proportion of infected nestlings was recorded in different species of birds [63,64,65]. However, chicks usually stay in the nests not for a long time, i.e., for about two to three weeks. The prolonged formation of sporozoites in mosquitoes may increase the gap between the availability of a host and the appearance of infective vectors, and therefore reduces the probability of successful parasite transmission. It is worth noting that additional information about the number of developed sporozoites and infectivity would greatly help to better understand the transmission of this parasite.

Another possible explanation for the absence of pGRW4 transmission in Northern Europe is that adult birds of genus *Acrocephalus*, which represent the main reservoir of *P. relictum* (pGRW4) in Europe, start migrating to their wintering grounds in Africa very early compared to the juveniles [66]. For example, autumn migration of Sedge warblers from Northern Europe begins in early July, and most adult birds leave their breeding sites by the middle of August [67]. On the contrary, the abundance of vectors from *Culex* genera peaks in August [68,69,70], including in Northern [70] and Southern Europe [68]. The prevalence of avian malaria parasites in mosquitoes reaches a maximum in July–August [69], or even in Autumn [68]. Apparently, most of the active transmission of avian malaria parasites in Europe occurs in the second half of the summer, when *Acrocephalus* birds are on their way to the breeding sites. Accordingly, the density of suitable mosquitoes is not sufficient to establish the transmission of the parasite. In southern regions, this gap between the availability of infected host and the maximum abundance of vectors could be shorter because of the longer presence of long-distance migrants. It is worth noting that in Southern Europe, *P. relictum* (pGRW4) was found in a single resident Blue tit (*Cyanistes caeruleus*) [71] and in a Cattle egret (*Bubulcus ibis*) [72] indicating the sporadic transmission of the parasite in this region.

Based on the results of this study, *C. pipiens* mosquitoes can serve as vectors of *P. relictum* (pGRW4). However, according to literature and the information available on the MalAvi database, pGRW4 was never found in this mosquito species, and the distribution of the parasite is associated with the presence of the natural vector—*C. quinquefasciatus* [11,30]. To clarify this, additional data from wild-caught vectors is needed.

According to several ecological studies, long-distance migrants caught in Europe demonstrate low intensities of pGRW4 parasitaemia [21,23,34]. These results are in accordance with experimental studies showing that even during primary infections, only light parasitaemia of *P. relictum* pGRW4 develop in the blood of some European species [29,73]. Additionally, a recent experimental study showed that during mixed infection with another malarial parasite, *P. relictum* (pSGS1), this parasite managed to develop only a transient infection, and disappeared when a sharp increase in pSGS1 infection was observed [74]. Coinfections are predominant in the wild, and interactions of different *Plasmodium* parasites can affect the development of one another [43,74,75], thus they should be taken into consideration in ecological studies as well. Unlike pGRW4, *P. relictum* pSGS1, which is naturally transmitted and prevalent throughout Europe, induces high parasitaemia in many vertebrate hosts [43] and potentially could suppress the tropical lineage.

The sporogonic development of malaria parasites in vectors is a complex process including changes in different stages from gametes to sporozoites. These transformations could be harmful to mosquitoes and reduce the vector’s longevity [76,77]. However, the accumulated data on the virulence of sporogonic stages to invertebrate hosts are contradictory, with some studies reporting the impact of malaria parasites on the vector to be low [76]. In our study, deaths of some exposed mosquitoes were observed in all experimental groups, although, significant differences between groups were not found. The times of death of all experimental mosquitoes were different (Table S1), and we did not detect increased mortality in a particular period of the sporogony, indicating the impact of the specific stages (migration of ookinetes through the midgut wall, formation of oocysts or migration of sporozoites to the salivary glands of the vector) on a vector’s survival.

*Culex pipiens* f. *molestus* were exposed to infected donors with lower and higher gametocytaemia. The load of sexual stages is one of the factors determining the sporogonic development of the pathogen [78], and is supposed to be associated with an infection burden in the invertebrate host [79]. In human and rodent malaria parasites, the number of ingested gametocytes by a mosquito is reflected in the rate of oocyst development [80,81,82]. However, similar correlations between gametocytaemia and the load of different sporogonic stages in vectors remain unclear in avian *Plasmodium* parasites. The experimental infection of *C. pipiens* mosquitoes with *P. relictum* (pSGS1) showed no positive associations between the oocyst burden and the level of gametocytes [83,84]. Similar results were obtained in the present study, where the number of the pGRW4 oocysts developed in mosquitoes did not depend on gametocytaemia in infected donor birds. Additionally, sporogony of all haemosporidians could be influenced by a complex of factors such as different maturity [85] and sex ratio of gametocytes [86], immune response of a vertebrate host and vector [79].

## 5. Conclusions

In conclusion, the obtained data show that the tropical, avian malaria parasite *P. relictum* (pGRW4) completes sporogony in *C. pipiens* f. *molestus* mosquitoes in Northern Europe, with night temperatures as low as 7 °C degrees. The exposure to the low temperature prolongs the time for oocyst and sporozoite development but does not terminate the sporogony completely. Low air-temperature leading to delays in *P. relictum* (pGRW4) sporogony in mosquitoes in the temperate zone may lead to interfered transmission of malarial parasites due to the lack of a vector with infective stages and vertebrate host at the same time and site. Together with other factors, such as transient infections and low competitiveness in coinfections, this may cause limitations of the transmission of pGRW4 lineage in Northern Europe. However, to fully understand the ecology and epizootiology of *P. relictum* (pGRW4), additional experimental and ecological studies are needed.

## Data Availability

The data presented in this study are available upon email inquiry.

## Supplementary Materials

The following are available online at https://www.mdpi.com/article/10.3390/microorganisms9112240/s1, Figure S1: The difference between *Plasmodium relictum* (pGRW4) oocyst numbers in *Culex pipiens* form *molestus* exposed to high and low intensity of gametocytemia. Table S1: Data of dissection of experimental mosquitos.
