# Supplementary material for: The Impact of Temperature on the Sporogonic Development of the Tropical Avian Malaria Parasite Plasmodium relictum (Genetic Lineage pGRW4) in Culex pipiens Form molestus Mosquitoes
Source: Microorganisms. 2021 Oct 28;9(11):2240. doi: 10.3390/microorganisms9112240 (PMC8620208; doi:10.3390/microorganisms9112240)
Supplement: Supplementary file 1 [file microorganisms-09-02240-s001.zip › microorganisms-1349641-supplementary.pdf]

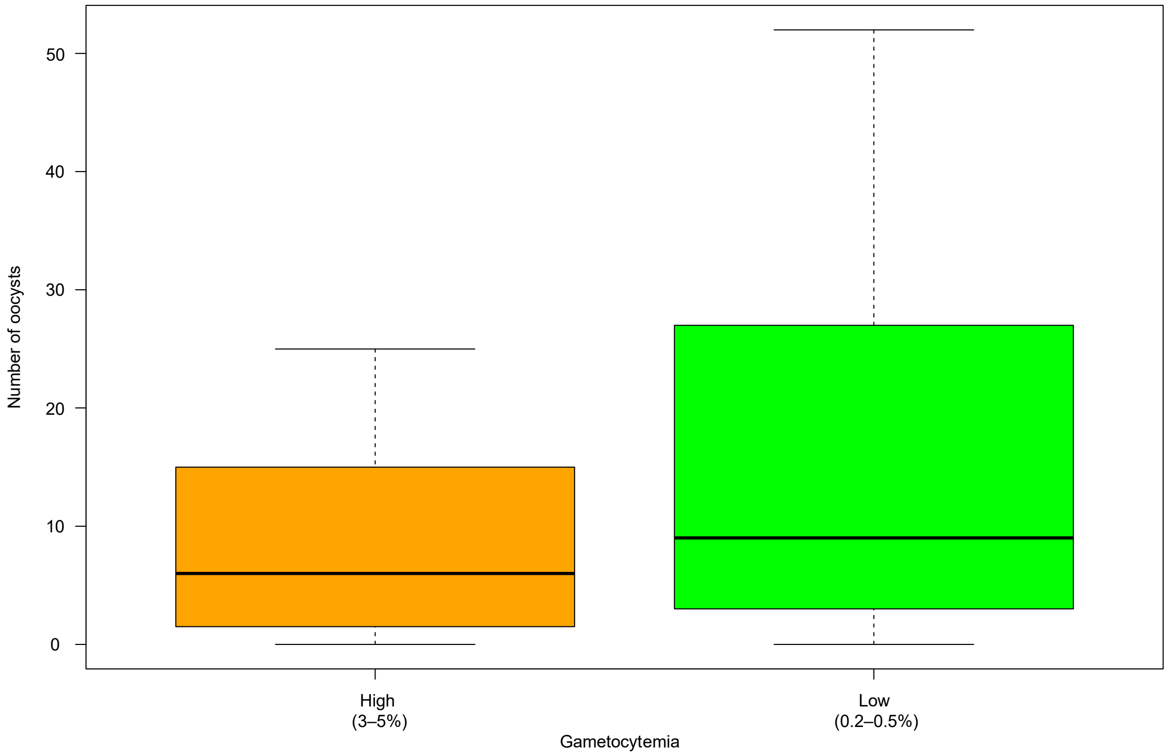

Figure S1: The difference between Plasmodium relictum (pGRW4) oocyst numbers in Culex pipiens form molestus exposed to high and low intensity of gametocytaemia.

Table S1. Dissection of experimental *Culex pipiens* form *molestus* mosquitoes exposed to avian malaria parasite *Plasmodium relictum* (genetic lineage pGRW4).

Number of oocysts	Presence of sporozoites	Days post exposure	Intensity of gametocytaemia	Experimental group	Mortality
N/A	N/A	2	Low	E1	Dead
N/A	N/A	4	Low	E1	Dead
13	N/A	6	High	E1	
7	N/A	6	High	E1	
23	N/A	8	High	E1	
N/A	N/A	8	High	E1	Dead
13	N/A	9	High	E1	Dead
44	N/A	10	Low	E1	
15	N/A	10	High	E1	
15	N/A	12	High	E1	
N/A	N/A	13	High	E1	Dead
N/A	N/A	14	High	E1	Dead
4	Positive	14	Low	E1	
136	Negative	14	High	E1	

N/A	N/A	15	High	E1	Dead
36	Positive	16	High	E1	
10	Negative	17	High	E1	Dead
9	Positive	18	Low	E1	
24	Positive	18	High	E1	
1	Positive	19	Low	E1	
11	Positive	20	Low	E1	
1	Positive	20	High	E1	
2	Positive	22	Low	E1	
2	Positive	22	High	E1	
2	Positive	22	Low	E1	
14	Positive	24	High	E1	
1	Positive	24	Low	E1	
7	Positive	26	Low	E1	
9	Positive	26	Low	E1	
0	Positive	28	Low	E1	
N/A	N/A	1	High	E2	Dead
N/A	N/A	2	High	E2	
N/A	N/A	2	Low	E2	
N/A	N/A	2	Low	E2	Dead
4	N/A	6	High	E2	
52	N/A	6	Low	E2	
40	N/A	7	Low	E2	Dead
N/A	N/A	8	High	E2	Dead
4	N/A	8	High	E2	
5	N/A	8	Low	E2	
N/A	N/A	9	High	E2	Dead
6	N/A	10	High	E2	
N/A	N/A	10	Low	E2	Dead
16	N/A	10	Low	E2	Dead
30	N/A	10	Low	E2	
7	N/A	10	High	E2	
26	N/A	10	Low	E2	
0	N/A	11	High	E2	
25	N/A	12	High	E2	
27	N/A	12	Low	E2	
36	N/A	12	High	E2	
80	N/A	12	Low	E2	
N/A	N/A	13	Low	E2	Dead
17	Negative	14	High	E2	
39	Negative	14	High	E2	
2	Negative	14	Low	E2	
19	Negative	14	Low	E2	
N/A	N/A	15	High	E2	Dead
N/A	N/A	16	High	E2	Dead

N/A	N/A	16	High	E2	Dead
25	Negative	16	High	E2	
3	Positive	16	Low	E2	
N/A	N/A	17	Low	E2	Dead
N/A	N/A	17	Low	E2	Dead
9	Positive	18	High	E2	
1	Positive	18	Low	E2	
2	Positive	20	High	E2	
10	Positive	20	Low	E2	
10	Positive	20	Low	E2	
27	Negative	20	Low	E2	
13	Positive	22	Low	E2	
9	Positive	22	Low	E2	
0	Positive	22	High	E2	
10	Positive	24	Low	E2	
6	Positive	24	Low	E2	
7	Positive	24	Low	E2	
2	Positive	24	High	E2	
7	Positive	24	High	E2	
0	Positive	26	High	E2	
14	Positive	26	Low	E2	
5	Positive	26	Low	E2	
0	Positive	28	High	E2	
1	Positive	28	Low	E2	
0	Positive	28	Low	E2	
N/A	N/A	2	High	E3	Dead
N/A	N/A	4	Low	E3	Dead
0	N/A	6	High	E3	
0	N/A	6	Low	E3	
7	N/A	6	Low	E3	
2	N/A	7	High	E3	
3	N/A	7	Low	E3	Dead
5	N/A	7	High	E3	
N/A	N/A	7	Low	E3	Dead
N/A	N/A	7	Low	E3	Dead
N/A	N/A	8	High	E3	Dead
1	N/A	8	High	E3	
2	N/A	8	High	E3	
0	N/A	8	Low	E3	
N/A	N/A	9	High	E3	Dead
36	N/A	10	Low	E3	
0	N/A	10	High	E3	
12	N/A	10	Low	E3	
17	N/A	12	High	E3	
49	N/A	12	Low	E3	

6	N/A	12	Low	E3	
16	N/A	12	Low	E3	
2	N/A	13	Low	E3	Dead
0	Negative	14	High	E3	
6	Negative	14	High	E3	
2	Negative	14	High	E3	
1	Negative	14	High	E3	
66	Negative	14	Low	E3	
1	Negative	14	Low	E3	
0	Negative	15	High	E3	Dead
21	Negative	15	High	E3	
0	Negative	16	High	E3	
7	Negative	16	Low	E3	
0	Negative	16	High	E3	
N/A	Negative	18	Low	E3	Dead
12	Negative	18	High	E3	
29	Negative	18	Low	E3	
10	Positive	20	High	E3	
50	Positive	20	Low	E3	
23	Negative	20	High	E3	
30	Positive	22	Low	E3	
4	Negative	22	High	E3	
12	Positive	24	Low	E3	
10	Positive	24	High	E3	
3	Positive	26	High	E3	
8	Negative	26	Low	E3	
N/A	N/A	27	Low	E3	Dead
12	Negative	28	High	E3	
